# 531. Clinical efficacy of inhaled corticosteroids in patients with coronavirus disease-2019: an updated systematic review and meta-analysis

**DOI:** 10.1093/ofid/ofad500.600

**Published:** 2023-11-27

**Authors:** Su Jin Jeong

**Affiliations:** Yonsei University College of Medicine, Seoul, Seoul-t'ukpyolsi, Republic of Korea

## Abstract

**Background:**

Inhaled corticosteroids (ICS) are known to be relatively safe for long-term use in inflammatory respiratory diseases, and it has been repurposed as one of the potential therapies for outpatients with coronavirus diseases 2019 (COVID-19). However, ICS have not been accepted for COVID-19 as a standard therapy because of its lack of proven benefits. Therefore, this study aimed to evaluate the effectiveness of ICS in patients with COVID-19.

**Methods:**

Randomized controlled trials comparing the efficacy of ICS treatment in patients with COVID-19 were identified through literature electronic databases searches up to March 10, 2023. Meta-analyses were conducted for predefined outcomes, and the certainty of evidence was graded using the grading of recommendations, assessment, development, and evaluation approach.

**Results:**

Overall seven trials (eight articles) were included in this systematic review. Compared with usual care, ICS was associated with significantly improved clinical recovery at 7 and 14 days in patients with COVID-19. In subgroup analysis, only budesonide showed significant efficacy in clinical recovery, whereas no significant benefit was observed for ciclesonide. Moreover, ICS use was not significantly associated with all-cause hospitalization, all-cause mortality, admission to intensive care unit, or the use of mechanical ventilation. Our systematic review used evidence with very low to moderate certainty.

**Figure:**
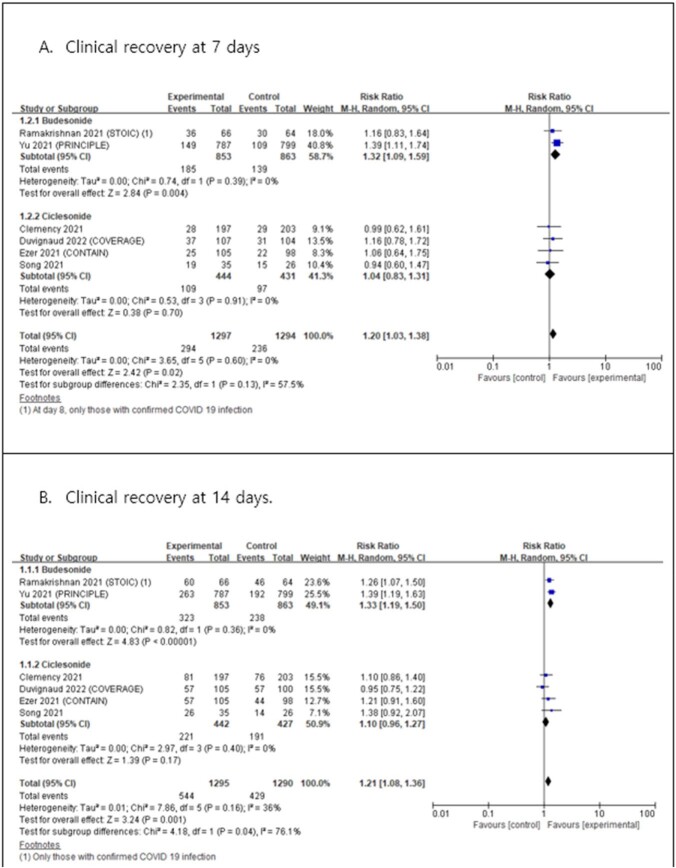

**Conclusion:**

The results of this meta-analysis revealed the clinical efficacy of ICS treatment compared with usual care. Based on limited evidence, our results suggest that ICS treatment, especially budesonide, improves the clinical recovery of patients with COVID-19. The safety of ICS treatment in patients with COVID-19 has not yet been established; however, ICS are relatively safe and widely available. In addition, short-term ICS use in the early stages of COVID-19 did not increase the risk of pulmonary infections or side effects. Subsequent randomized clinical trials with a larger number of patients, as well as meta-analyses, are needed to determine the usefulness of ICS treatment in improving the clinical outcomes of patients with COVID-19.

**Disclosures:**

**All Authors**: No reported disclosures

